# Pore Structure and Synergy in Antimicrobial Peptides of the Magainin Family

**DOI:** 10.1371/journal.pcbi.1004570

**Published:** 2016-01-04

**Authors:** Almudena Pino-Angeles, John M. Leveritt, Themis Lazaridis

**Affiliations:** Department of Chemistry, The City College of New York, New York, New York, United States of America; University of Illinois, UNITED STATES

## Abstract

Magainin 2 and PGLa are among the best-studied cationic antimicrobial peptides. They bind preferentially to negatively charged membranes and apparently cause their disruption by the formation of transmembrane pores, whose detailed structure is still unclear. Here we report the results of 5–9 μs all-atom molecular dynamics simulations starting from tetrameric transmembrane helical bundles of these two peptides, as well as their stoichiometric mixture, and the analog MG-H2 in DMPC or 3:1 DMPC/DMPG membranes. The simulations produce pore structures that appear converged, although some effect of the starting peptide arrangement (parallel *vs*. antiparallel) is still observed on this timescale. The peptides remain mostly helical and adopt tilted orientations. The calculated tilt angles for PGLa are in excellent agreement with recent solid state NMR experiments. The antiparallel dimer structure in the magainin 2 simulations resembles previously determined NMR and crystal structures. More transmembrane orientations and a larger and more ordered pore are seen in the 1:1 heterotetramer with an antiparallel helix arrangement. Insights into the mechanism of synergy between these two peptides are obtained via implicit solvent modeling of homo- and heterodimers and analysis of interactions in the atomistic simulations. This analysis suggests stronger pairwise interactions in the heterodimer than in the two homodimers.

## Author Summary

The emergence of antibiotic resistance has created a compelling need for new potent antibiotics. Antimicrobial peptides, naturally produced by many organisms, have long been pursued as a means to fill this gap. However, they have not yet realized their practical potential, partly due to a lack of full understanding of their mechanism of action. The formation of pores by these peptides in bacterial membranes has been demonstrated by multiple biophysical approaches, but the detailed structure of these pores is still unclear. The magainin family of antimicrobial peptides have been thoroughly studied over the last three decades. We have generated atomic-resolution models of membrane pores generated by tetrameric assemblies of different magainin peptides by means of molecular dynamics simulations on the multimicrosecond timescale. The results complement previous experimental findings and set the stage for further development of theoretical and experimental approaches that may ultimately allow the rational design of improved antibiotics.

## Introduction

The magainin family of antimicrobial peptides are found in the skin of the frog *Xenopus laevis* [1]. Magainin 2 (MAG2) and PGLa are the best studied peptides in this family. They are active against a broad range of microorganisms [1,2] and exhibit cytolytic effects on certain tumor cell lines [3,4]. They are unstructured in solution but fold as amphipathic α-helices in the presence of TFE and upon binding to lipids [5–7]. Like other antimicrobial peptides, they have been found to act on membranes [8–10], presumably forming pores [11]. Despite intensive experimental efforts, the detailed structure of these pores is still unknown.

MAG2 has been studied extensively for almost 30 years now. It binds to the surface of anionic lipid bilayers mainly through electrostatic interactions, where it causes thinning and, eventually, disruption of the membrane potential and cell lysis [1,12]. At high peptide concentration a portion of the peptides undergo a transition from surface to transmembrane orientation [13,14]. Results from in-plane neutron scattering experiments in oriented multilayers showed that an average of 4 to 7 MAG2 peptides stabilize toroidal pores of 70 Å outer diameter [14]. These results were further corroborated by cryo-EM, which showed that MAG2 forms pores of ~80 Å diameter in DMPC/DMPG vesicles [15]. It has been observed that the pores do not affect significantly the integrity of the membrane [16,17]. Upon the eventual pore closure, the peptides translocate to the inner leaflet to balance the peptide concentration in each leaflet [18].

PGLa is a similar peptide produced in the glands of the same frog [19], which has been found to promote membrane permeabilization [20,21]. Experimental evidence has suggested that PGLa probably forms short-lived pores as a consequence of the instability introduced by the electrostatic repulsions between its positively charged residues [10]. Like MAG2, PGLa translocates from the outer to inner membrane leaflets, induces lipid flip-flop, alters membrane curvature and promotes the formation of peptide-lipid clusters [22]. Solid-state NMR studies have provided information on the orientation of the peptides in membranes with different lipid composition. At low peptide to lipid molar ratio (P/L) PGLa adopts an orientation parallel to the membrane surface (S-state) [23], whereas at higher P/L it adopts intermediate, tilted orientations (T-state) [24].

Apart from their individual activity, the combination of MAG2 and PGLa results in enhanced cytolytic, antitumor and antibacterial effects [25,26]. Fluorescence and dye-leakage experiments showed that the mixture shares the same mechanism of action as the individual peptides and exhibits faster pore formation but moderate pore lifetime [10]. The best synergistic performance is achieved in 1:1 stoichiometric mixtures, which suggests that MAG2 and PGLa may form heterodimers. Single-site mutations decreased the synergy, supporting the idea of specific interactions [10]. A cross-linking study suggested a parallel topology for the heterodimer [27]. Solid state NMR studies showed that the presence of MAG2 increases the probability of a fully inserted state (I-state) for PGLa [28] but MAG2 remains parallel to the membrane surface [29]. It was suggested that MAG2 may facilitate the insertion of PGLa by thinning the bilayer [29] but more recent work showed that the orientation of PGLa depends on lipid spontaneous curvature rather than thickness [30].

As in other biophysical problems, molecular modeling and computer simulations have the potential to provide atomic level insights. Spontaneous generation of a toroidal pore was first described in an atomistic molecular dynamics simulation by Marrink's group [31]. This work studied a tetramer of a magainin derivative that is more hydrophobic and thus active on neutral membranes (MG-H2 [32]). The authors found that the MG-H2 peptides were extensively unfolded and located mostly on the rim of the water channel. This arrangement was termed a “disordered toroidal pore”. Further all-atom simulations and longer time-scale simulations at the coarse-grained level reaffirmed the formation of a disordered toroidal pore by MG-H2 [33]. The role of charge distribution on pore formation was addressed in atomistic simulations starting from a transmembrane bundle [34]. That study showed that the positive charges in the N-terminus of MG-H2 (residues K3 and K4) are critical for the formation of a toroidal pore, whereas positive charges in the middle of the helix (residue K11 and K14) introduce more disorder into the pore structure. More recent coarse-grained simulations revealed a mechanism of creating giant pores via membrane remodeling [35] and the formation of large clusters and disordered toroidal pores [36]. The mechanism of PGLa-magainin synergy has also been explored with coarse-grained simulations [37].

In the present work, we have investigated the mechanism of pore stabilization by magainin peptides via 5–9 μs all-atom molecular dynamics simulations on the Anton supercomputer. Since the time scale for pore formation is unknown (likely longer than the timescales currently accessible), these simulations started from tetrameric helical bundles inserted in a transmembrane orientation. The rationale was that the local free energy minima corresponding to peptide-stabilized membrane pores should be closer to this starting condition than to peptides adsorbed on the membrane surface, so that simulations could hopefully reach them on the above timescale. Simulations were performed for MAG2 and PGLa homotetramers, as well as 2:2 heterotetramers. Antiparallel arrangements of the helices were also simulated, based on implicit solvent modeling results showing antiparallel dimers to be more favorable. For each system, the membrane composition was chosen according to the optimal activity of the peptide observed *in vitro* [10,14,21,32]. The results are compared to the available experimental data and provide insights into the mechanism of membrane pore stabilization by these peptides at the atomic level.

## Results

In this work six tetramers of magainin-family peptides were simulated on the Anton supercomputer for 5 to 9 μs starting from membrane-inserted helix bundles (Table 1). Lipid composition, membrane patch size, starting helix orientation, and peptide bundle composition were varied to examine the effect of these parameters and to obtain insights into the observed synergy between the two peptides. In all the simulated systems the peptides rapidly reoriented, allowing the penetration of water molecules in the bilayer and formation of a channel. However, the final orientation of the peptides and the size of the water pore differed significantly among the six systems. Although the spatial distribution of the peptides around the pore varies between systems, all pores are stabilized by the four monomers, which remain inside the membrane for the whole simulation (S1 Fig). A detailed description of the simulations is given in this section and comparison with experimental data and previous studies is done in the Discussion. In what follows, we have taken into consideration the terminology used in previous studies [30] and will refer to tilt angles in the range 60°-120° as S-state, 30°-60°/120°-150° as T-state, and 0–30°/150-180° as I-state.

**Table 1:** Systems simulated^a^.

	Peptide initial orientation	Membrane composition	Number of lipids^b^	Simulation length	P/L ratio
MAG2	Parallel	DMPC/DMPG 3:1	80	9 μs	1:20
MAG2	Antiparallel	DMPC/DMPG 3:1	120	5 μs	1:30
PGLa	Parallel	DMPC/DMPG 3:1	120	5 μs	1:30
MG-H2	Parallel	DMPC	71	9 μs	~1:18
PGLa-MAG2	Parallel	DMPC	71	9 μs	~1:18
PGLa-MAG2	Antiparallel	DMPC/DMPG 3:1	120	9 μs	1:30

^a^ Peptide sequences: MAG2: GIGKFLHSAKKFGKAFVGEIMNS; PGLa: GMASKAGAIAGKAIAK VALKAL-NH2; MG-H2: IIKKFLHSIWKFGKAFVGEIMNI

^b^ Approximate cell sizes: 56x56x60 Å (71–80 lipids) and 66x66x74 Å (120 lipids).

### MAG2 parallel tetramer in 80-lipid DMPC/DMPG

**Fig 1:**
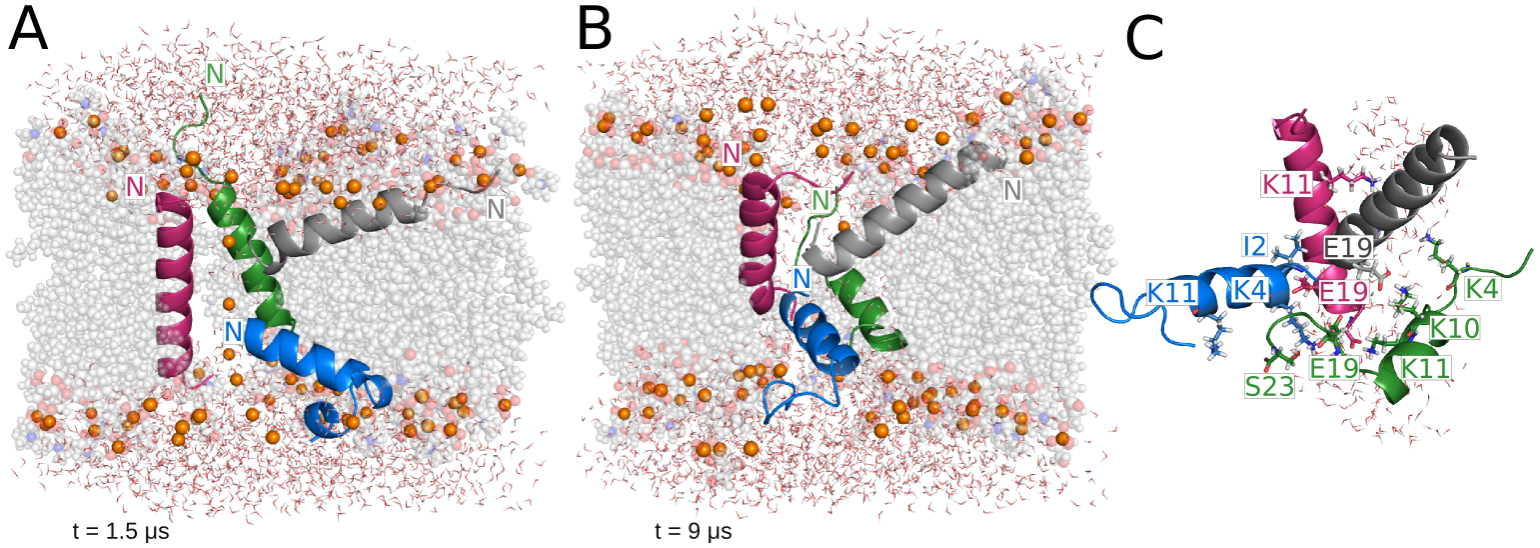
Representative structures from the MAG2 parallel tetramer simulation. (A) Thin pore supported by an irregular distribution of MAG2 monomers. (B) Final structure. (C) Amino acids involved in intermolecular interactions formed during the last microsecond of the trajectory and pore waters. In all the figures, lipids are shown as grey spheres, phosphorus atoms representing the lipid headgroups are shown as orange spheres and water molecules as lines. Lipid spheres are semi-transparent and some of the molecules have been omitted for clarity.

**Fig 2:**
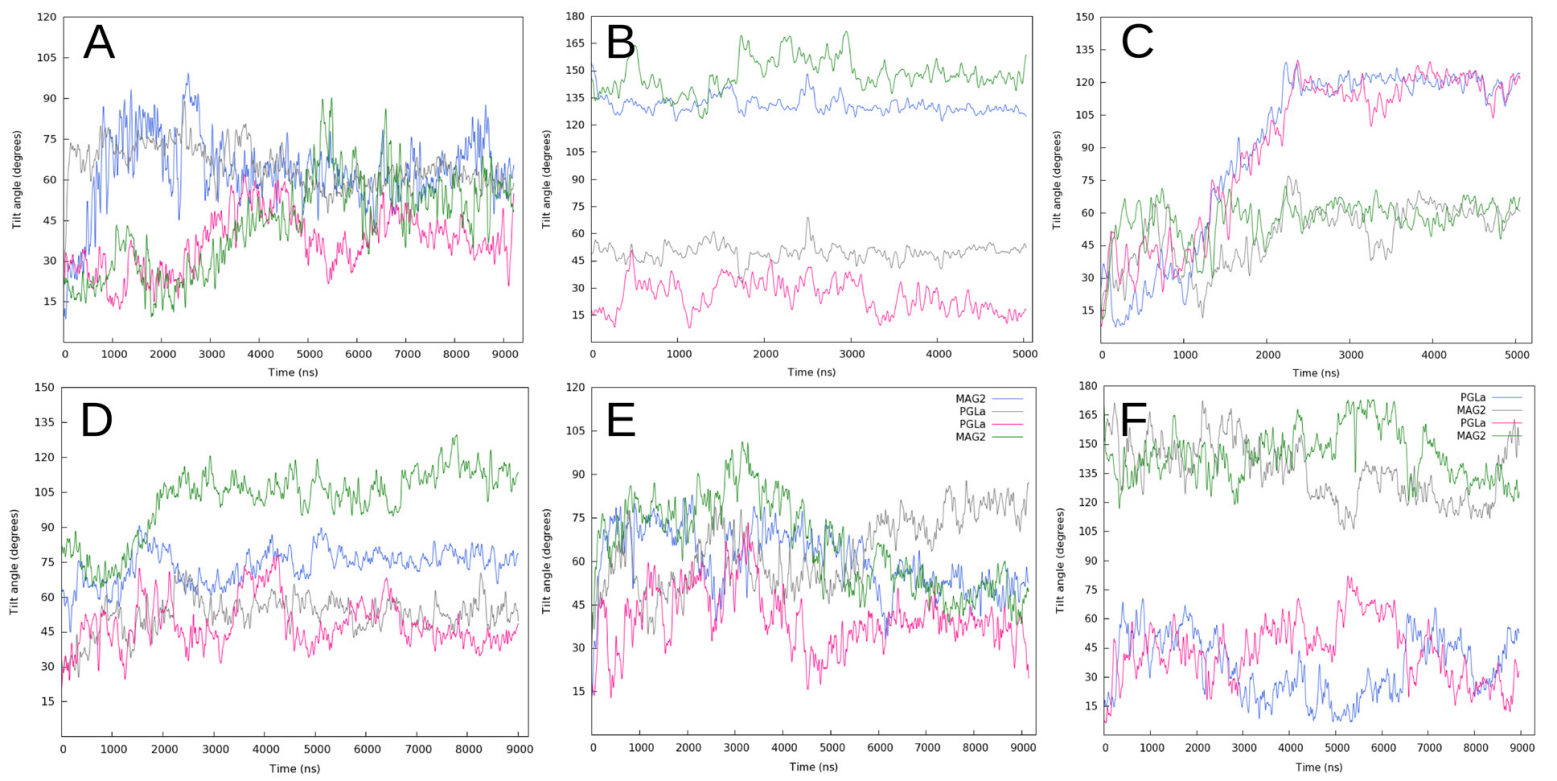
Peptide orientation changes in the membrane. The plot shows the tilt angle values for MAG2 in (A) 80 lipids and (B) 120 lipids, PGLa (C), MG-H2 (D), and PGLa-MAG2 in DMPC (E) and DMPC/DMPG (F) simulations. The colors on this plot correspond to the colors of the peptides in Figs 1 and 4–8.

The initial structure in this simulation is a tightly packed, parallel 4-helix bundle (N terminus in the "upper" leaflet in Fig 1). During the first microsecond, two of the monomers adopt S-state orientations on opposite leaflets, while the remaining two peptides remain in a slightly tilted T-state (Fig 1A). As a result of the reorientation, the monomer located on the upper leaflet inserts its C-terminus deeper into the membrane, whereas the peptide on the lower leaflet inserts its N-terminus deeper. During these first steps of the simulation, several lipids bend and insert their headgroups into the membrane and around the channel. This configuration is fairly stable during the first 3 μs of the trajectory. At that point, the four monomers insert their terminal regions deeper into the membrane and adopt T-state orientations with average tilt angles from 45–60° (Figs 1B and 2A). This configuration, which does not change significantly for the remaining 6 μs of the simulation, is supported by an extensive set of pairwise intermolecular interactions (Fig 1C). The orientation of the monomers favors interactions between residues located in the N- and C-terminal regions. During the last microsecond of the trajectory, the interacting network is mainly comprised of the charged residues E19, K4, K10 and K14, and also S23 in the C-terminus. As a result, neighboring monomers have interaction energies ranging from -120 to -200 kcal/mol.

**Fig 3:**
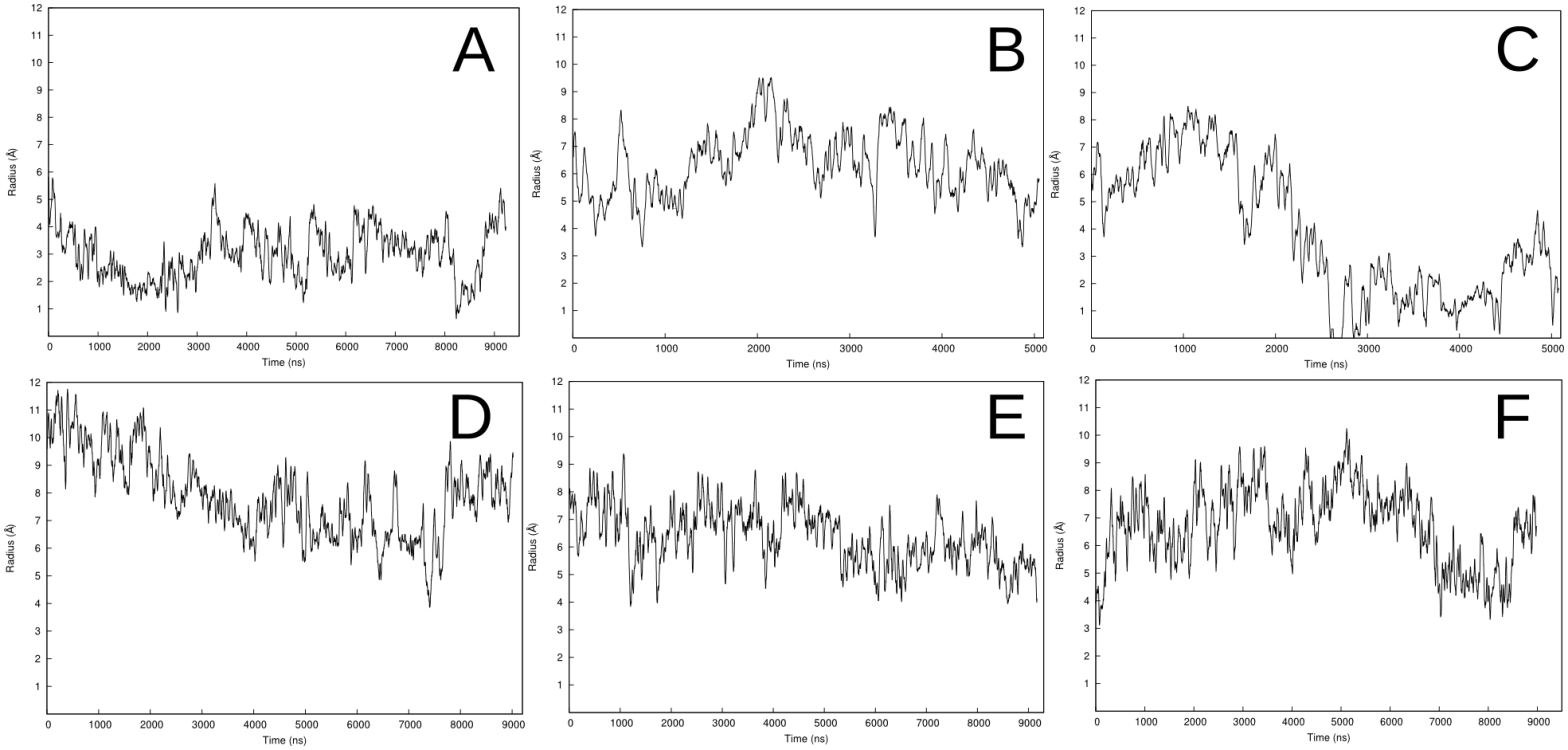
Evolution of the transmembrane pore. The plot shows the inner pore values variation for MAG2 in (A) 80 lipids and (B) 120 lipids, PGLa (C), MG-H2 (D), and PGLa-MAG2 in DMPC (E) and DMPC/DMPG (F) simulations.

In this stable configuration, the terminal regions of the four peptides (2 C- and 2 N-termini) are located in very close proximity. As a consequence, the pore radius decreases in the last part of the trajectory, and we observe that the peptides frequently block the channel and interrupt the water flow (Fig 3A). As a result of the tight structural arrangement, only 3 lipid headgroups on average penetrate in the hydrophobic area of the bilayer during the last microsecond of the trajectory (Table 2).

**Table 2:** Structural analysis of the transmembrane pores. The values correspond to averages over the last microsecond of the trajectory.

	Helicity*	Tilt angle*	Number of headgroups	Number of water molecules^+^	Inner pore radius
MAG2, 80-lipids	52-81-69-41	67-59-36-54	3 ± 1	1469 ± 114	3 ± 1.5
MAG2, 120 lipids	85-84-78-77	128-51-18-147	7 ± 2	1582 ± 220	5.6 ± 1
PGLa	90-90-90-90	120-59-119-61	5 ± 2	1215 ± 241	2.3 ±1.2
MG-H2	81-77-85-67	76-55-42-113	5 ± 1	1017 ± 187	8 ± 0.9
PGLa-MAG2, 71 lipids	59-85-82-84	51-80-35-49	3 ± 1	791 ± 116	5 ± 0.9
PGLa-MAG2, 120 lipids	89-82-77-82	36-133-25-130	8 ± 2	1556 ± 232	5.6 ± 1.4

**Fig 4:**
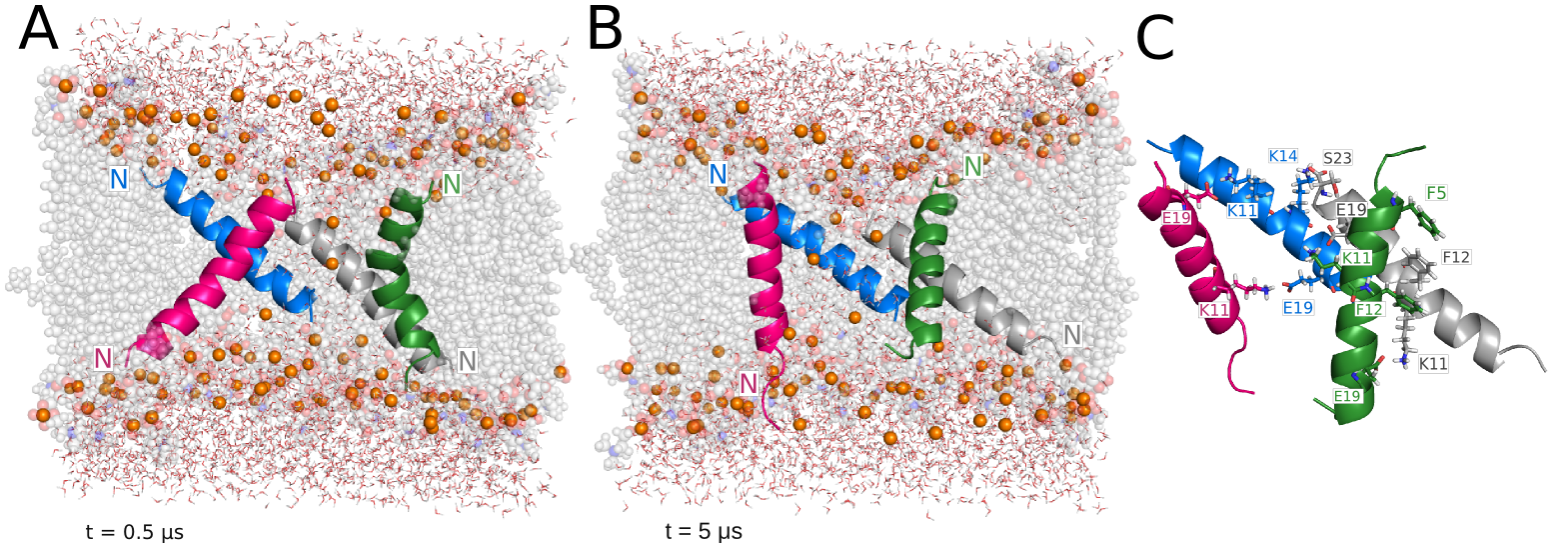
Representative structures from the MAG2 antiparallel tetramer simulation. (A) Monomers in T-state aligned around a wide water channel. (B) Final structure of the pore supported by two magainin peptides in the I-state. (C) shows the individual amino acids involved in the intermolecular interactions formed during the last microsecond of the trajectory.

**Fig 5:**
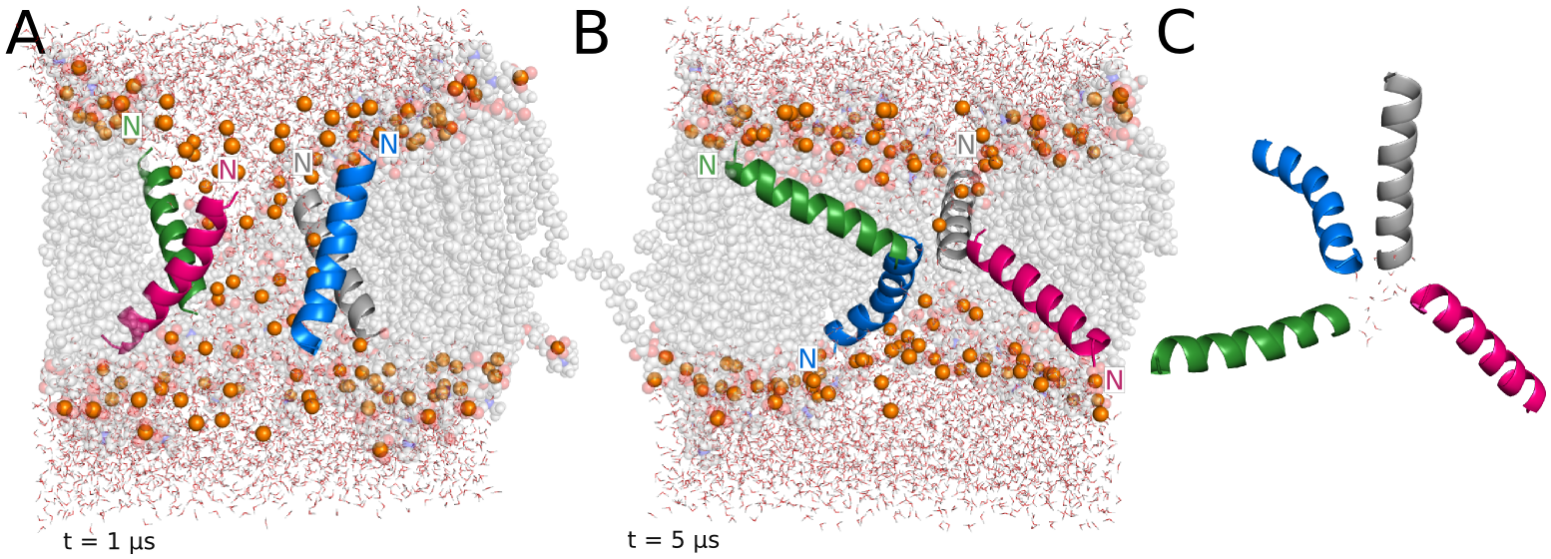
Representative structures from the PGLa tetramer simulation. (A) Wide toroidal pore comprised by the peptides and multiple lipid molecules at the beginning of the simulation. (B) Final structure of the tetramer around a very thin water channel. (C) Top view of the final structure of the PGLa tetramer.

**Fig 6:**
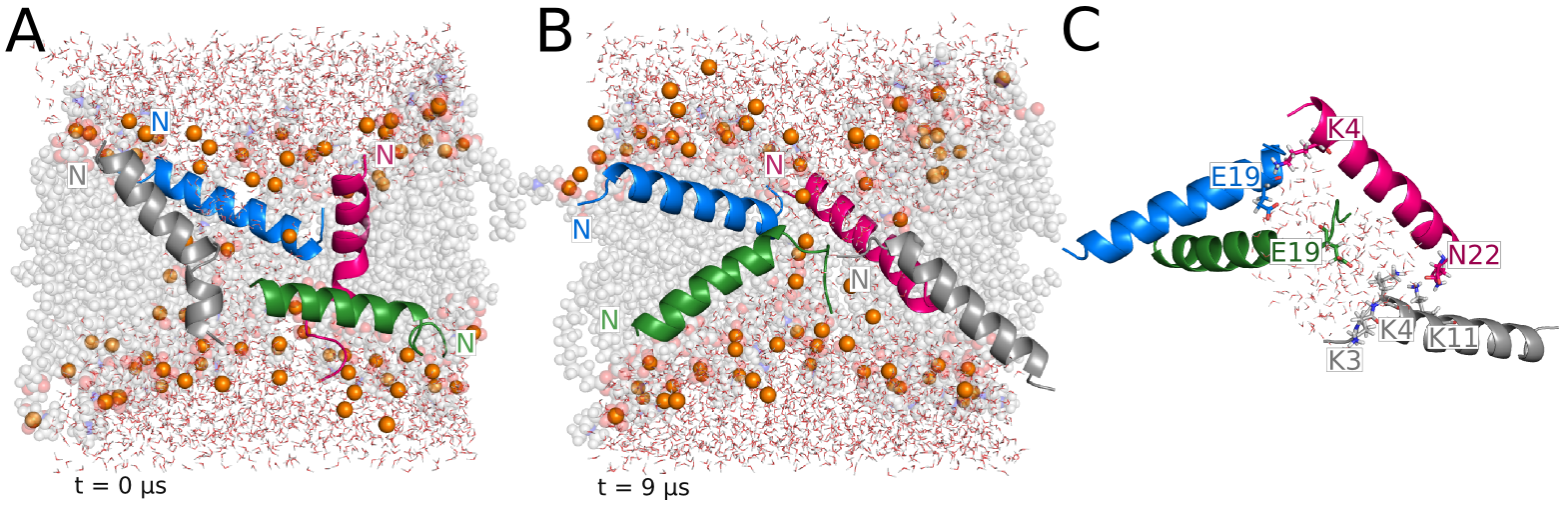
Representative structures from the MG-H2 tetramer simulation. (A) Starting configuration corresponding to the last frame of the simulation by Mihajlovic and Lazaridis [34] and (B) final structure showing a disordered wide pore, stabilized by MG-H2 and multiple lipid molecules. (C) shows the individual amino acids involved in the intermolecular interactions formed during the last microsecond of the trajectory.

**Fig 7:**
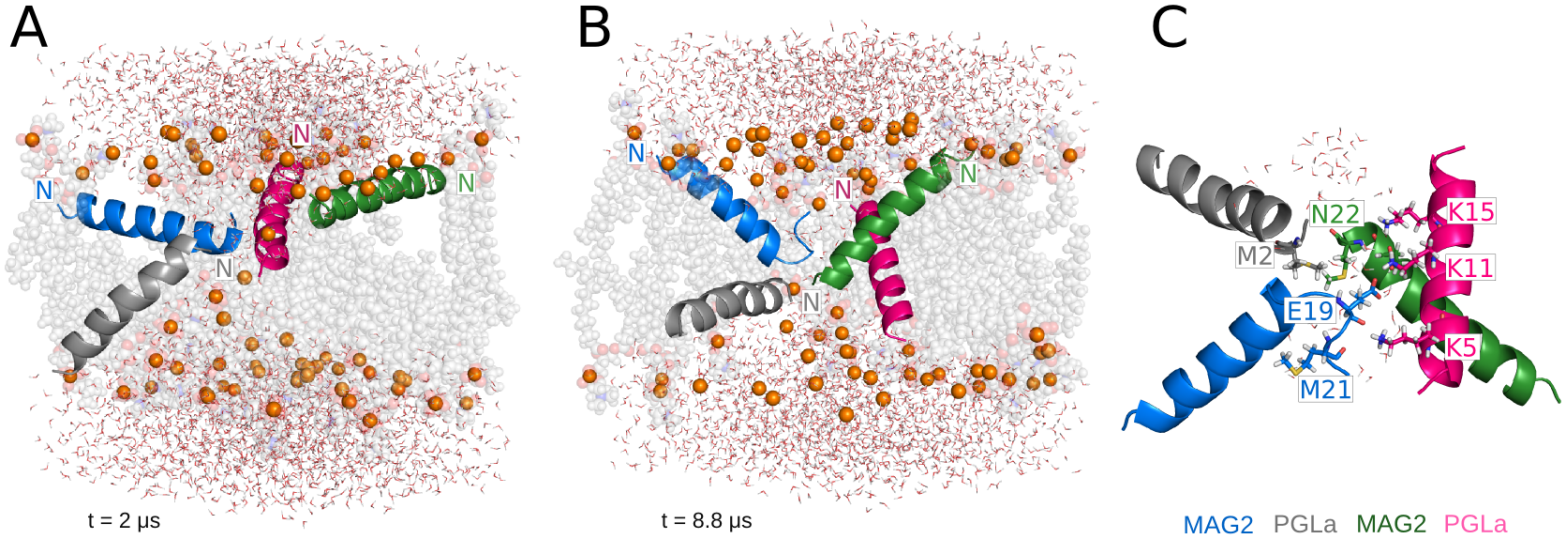
Representative structures from the PGLa-MAG2 parallel tetramer simulation. Intermediate (A) and final structures (B) obtained from the PGLa-MAG2 tetramer simulation in a DMPC membrane. (C) shows the individual amino acids involved in the intermolecular bonds formed during the last microsecond of the trajectory and pore waters. The legend in the right bottom corner shows the color code for each peptide.

**Fig 8:**
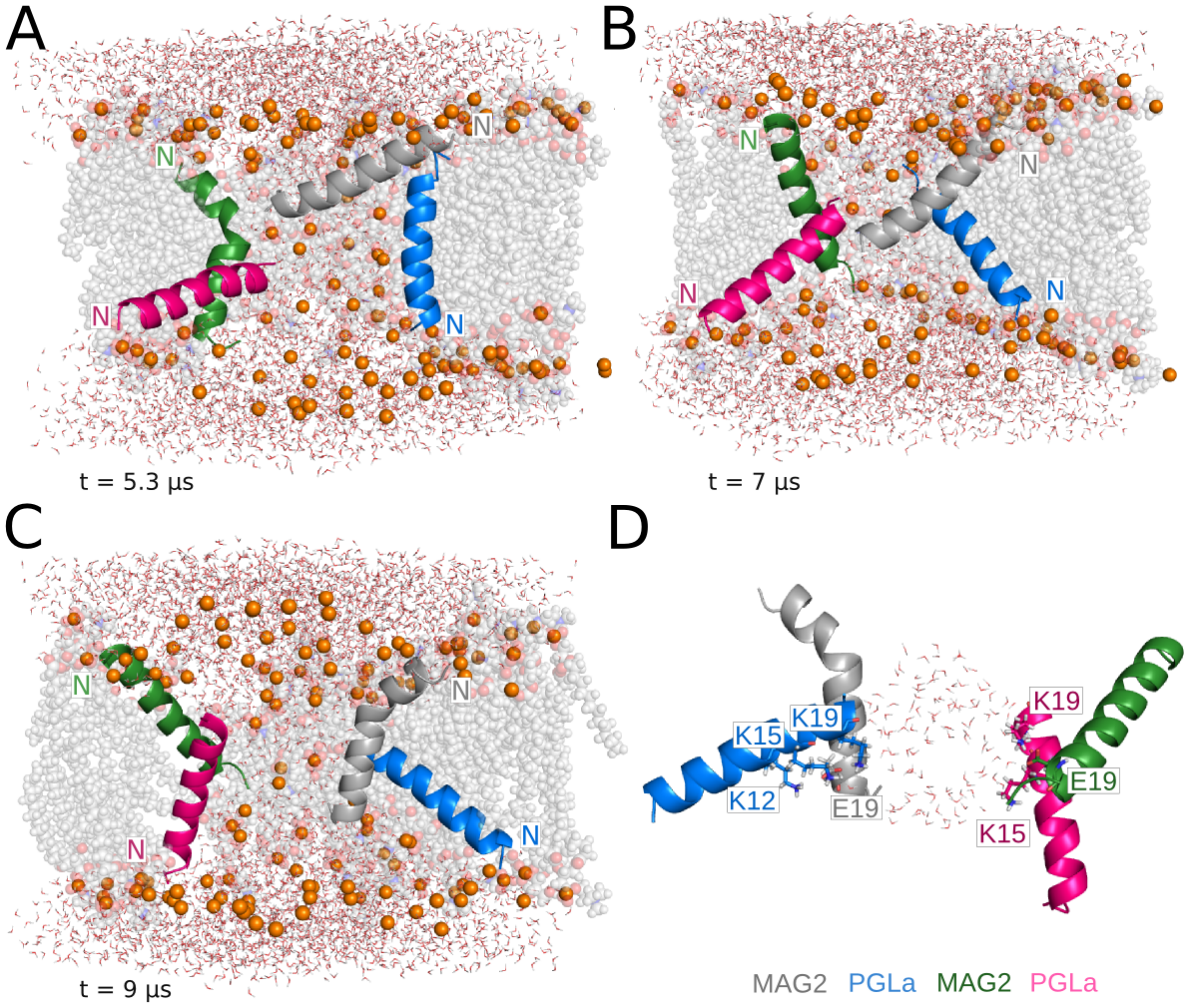
Representative structures from the PGLa-MAG2 antiparallel tetramer simulation. (A) Two S-state, two I-state configuration moves to (B) an intermediate conformation of mostly T-state, to a final (C) two tilted S-state, two I-state configuration. (D) shows the amino acids involved in the intermolecular interactions formed during the last microsecond of the trajectory and pore waters.

* The values correspond to peptides blue-grey-pink-green in Figs 1 and 4–8, respectively.

### MAG2 antiparallel tetramer in 120-lipid DMPC/DMPG

The initial configuration of this system consisted of two antiparallel dimers placed parallel to each other in a transmembrane orientation with the hydrophobic face outwards. This choice was motivated by implicit membrane modeling results of the possible combinations of dimer orientations on the membrane surface, in which the antiparallel one was found to be the most energetically favorable (see S1 Text).

A wide water channel opens during equilibration, reaching ~7 Å radius at the beginning of production (Fig 3B). In the first microsecond of the simulation, the four monomers adopt a tilted orientation (Fig 4A). Two of the peptides, colored blue and grey in Fig 4, maintain the adopted T-state conformation for the rest of the trajectory, with very stable average tilt angle values of 131° ± 6 and 50° ± 6, respectively (Fig 2B). The other two monomers adopt a dynamic equilibrium between T-state and I-state configurations from 2 μs to the end of the 5 μs simulation. At the end of the trajectory, these two monomers stabilize in a transmembrane orientation, with average tilt angles during the last microsecond of 147° ± 6 and 18° ± 6.

The final configuration of the tetramer shows a symmetrical arrangement of the monomers in two well defined T-state and two inserted orientations (Fig 4B). The original antiparallel dimers remain associated but with a nonzero crossing angle. This structure of the dimers is stabilized by multiple interactions between charged groups and the stacking of Phe residues (Fig 4C). Interestingly, it resembles the available NMR structure [38] and a crystal structure of a magainin variant [39], although the crossing angle in the latter is smaller (S2 Fig). The monomers in contact have average interaction energies of -223 ± 45 kcal/mol (blue and grey peptides in Fig 2), -166 ± 47 kcal/mol (green and grey peptides), and -78 ± 48 kcal/mol (pink and blue peptides) during the last microsecond of the simulation. Although the alignment of the peptides varies during the trajectory, the overall configuration of the pore structure is fairly stable. The pore is further supported by a variable number of headgroups that surround the channel between the two I-state monomers (Fig 4B). The amount of headgroups lining the pore walls depends greatly on the pore size along the trajectory. The channel radius fluctuates around an average value of ~6 Å to maximum peaks between 7–8 Å (Fig 3B).

### PGLa parallel tetramer in 120-lipid DMPC/DMPG

The pore formed during equilibration increases in size up to 6–7 Å radius during the first two microseconds (Figs 5A and 3C). In this initial period, all the monomers explore different transmembrane and surface orientations, and an average of 6–8 headgroups penetrate into the bilayer to line the channel. At ~1.5 μs two of the monomers transition to an S-state conformation on the surface of the lower leaflet (peptides colored blue and pink in Fig 5), and they move across the periodic images at different moments in the trajectory. During these transitions, the channel is supported by the three remaining monomers, until the tetrameric structure is restored between 2 and 2.5 μs. The tetrameric assembly is then stable for the rest of the trajectory, and displays the four monomers in tilted T-states around a very thin water channel (Fig 5B). For the last 2.5 μs, the monomers in the upper and lower leaflets have almost identical tilt angle values of 57.7° ± 7.7 and 60.6° ± 6.0, and 120.0° ± 5.0 and 117.6° ± 7.0, respectively (Fig 2C).

This final configuration promotes a drastic decrease of the size of the pore, which is even transiently blocked at ~3 μs (Figs 5B and 3C). During the last two microseconds of the simulation, the average pore radius is 2.4 ± 1.2 Å. The number of headgroups involved in pore stabilization also decreases during the last microsecond of the trajectory to an average of 5 (Table 2). The simulation was stopped at 5 μs due to the lack of any significant changes in the structure of the tetramer and the small diameter of the pore. Despite their proximity, none of the peptides form specific interactions, which translates to insignificant interaction energy values between monomer pairs.

### MG-H2 parallel tetramer in 71-lipid DMPC

This simulation extends a previous 160-ns one in which the peptides organized around a disordered toroidal pore, from an initial transmembrane orientation [34]. The starting configuration in the present study includes two monomers in a T-state and two S-state located on opposite leaflets (Fig 6A). The four monomers are highly mobile throughout the trajectory, leading to major variations in the pore structure. During the first ~2 μs, the peptides exchange between different orientations inside the membrane, until they reach a stable conformation that is maintained until the end of the simulation (Fig 6B). Here also we observed the translation of one monomer into the neighboring periodic image (peptide colored green in Fig 6). During this transition, the pore was stabilized by the three remaining peptides and by lipid headgroups, until the monomer translated completely and re-established the tetrameric structure. This transient event did not affect the integrity of the pore. Afterwards, the tetramer adopts a stable configuration in which the monomers arrange in pairs, facing opposite terminal regions towards the water channel (Fig 6B). This spatial configuration allows the formation of intermolecular interactions between charged side chains in the N- and C-terminal regions (Fig 6C) that contribute to favorable interaction energy only between peptides in the same pair (-324 kcal/mol ± 71 for peptides grey and green in Fig 4, and -186 kcal/mol ± 45 for peptides blue and pink).

The pore size decreases gradually during the simulation to eventually grow up again to a final value of 8.2 Å ± 0.9 during the last microsecond (Fig 3D). Sharp alterations in the pore radius values are the result of the translation event and the partially unfolded regions of two of the monomers. These regions comprise most of the charged amino acids involved in the formation of salt bridges, which is favored by the flexibility provided by the absence of a defined secondary structure. The average helicity during the simulation ranges between 67–82%, which is significantly higher than that reported in a previous computational study [31].

### PGLa-MAG2 parallel tetramer in 71-lipid DMPC

This heteromer is initially assembled in a parallel orientation with the N-termini of all the monomers pointing towards the upper leaflet. The monomers sample multiple orientations during the first half of the trajectory. At ~2 μs, the two MAG2 peptides and one of the PGLa monomers pull their C-termini towards the surface of the upper leaflet, adopting a highly tilted orientation (Fig 7A). The remaining PGLa monomer also increases its tilt angle by bending its N-terminus to the water channel. The three monomers on the upper leaflet rapidly transition to a T-state conformation. At ~6.5 μs the tetramer reaches a stable configuration comprised by the two MAG2 monomers with their C-termini buried in the bilayer, a PGLa monomer in a tilted T-state, and the remaining PGLa peptide almost parallel to the lower leaflet, with an average tilt angle of 80° ± 5 (Figs 7B and 2E). The average tilt angles of the three transmembrane monomers during the last microsecond range between 35° to 50° (Fig 2E).

The size of the pore varies abruptly for 6 μs together with the exchange in transmembrane orientations of the four monomers (Figs 2E and 3E). The sharp changes in pore size observed from ~6 to 9 μs are due to a partial blockage of the channel by the unfolded C-terminus of one of the MAG2 monomers (peptide colored blue in Fig 7). The helicity of the fragment between residues V17 to S23 drops to almost 0% for the last three microseconds of the trajectory (Table 2). The increased flexibility of this unfolded region favors the approach of E19 to the lysine residues in the neighbor PGLa monomer, hence obstructing the pore. Nevertheless, the channel is continuously open with an average pore radius 6.4 Å ± 1.2 for the entire simulation.

The two MAG2 monomers interact individually with the PGLa monomer in the T-state through charged residues (Fig 7C). The interaction energies of these PGLa-MAG2 pairs during the last microsecond are -211 ± 57 and -225 ± 37 kcal/mol. The PGLa in the tilted S-state interacts exclusively with one MAG2 monomer through M2 and N22, respectively. The interaction energy of this pair is -126 ± 52 kcal/mol. Interaction energies between MAG2-MAG2 and PGLa-PGLa pairs are negligible.

### PGLa-MAG2 antiparallel tetramer in 120-lipid DMPC/DMPG

The antiparallel dimer structure resulting from the implicit membrane calculations (see S1 Text) was duplicated and arranged as a transmembrane tetramer. A water channel opens during equilibration and rapidly increases in size (Fig 3F). As a result of pore growth, the tetramer splits into two heterodimers that do not interact with each other but display similar dynamic behavior during the entire simulation. For the first 5 μs, the four monomers transition between T-state conformations. At this point, a MAG2 and a PGLa of opposite heterodimers adopt an almost perfect I-state conformation, whereas the other two monomers move towards the upper and lower leaflet of the membrane (Fig 8A). The intermolecular interactions are reduced to transient salt bridges between E19 of MAG2, and K12, K15 and K19 of PGLa in just one of the heterodimers (monomers colored gray and pink in Fig 8). At ~6 μs, the four monomers adopt tilted transmembrane orientations (Fig 8B) that result in a significant decrease in the pore size (Fig 3F). During the last microsecond, the pore size increases again as the heterodimers separate into a configuration that resembles the one previously observed at ~5 μs, but with the two peptides in each dimer having roughly exchanged orientations (Fig 8C). In this last structure, the interface area between monomers is wider, and the charged side chains of both heterodimers participate in the formation of intermolecular interactions (Fig 8D). The average interaction energies for the last microsecond for the heterodimers are -151 ± 50 and -210 ± 63 kcal/mol. The large pore is stabilized by an average of 8 headgroups inserted in the hydrophobic region of the bilayer during the last microsecond of the trajectory, although we observed peaks of 10–12 headgroups during the pore expansion period at 4.5 μs (Table 2).

## Discussion

The all-atom simulations reported here are, to our knowledge, the longest yet on antimicrobial peptides. In these simulations four parameters were varied: membrane patch size, membrane composition, peptide bundle composition, and starting arrangement (parallel or antiparallel). The limited computational resources did not allow us to try all possible combinations of these four parameters to assess the effect of each one individually. Another important parameter that was not varied was oligomeric number; only tetramers were considered here based on experimental suggestions [8,14,40]. In most instances the pore structures seem converged, based on the lack of further changes near the end of the simulation. However, the effect of the initial peptide arrangement is not fully eliminated on this timescale. This is most clearly seen in simulations 1 and 2, where the same peptide bundle is simulated in the same lipid composition but with different starting arrangements and different box size giving a distinct final pore structure. Despite these limitations, important insights have been obtained by these simulations which help interpret some key experimental data, as discussed below.

Experimental results on pore size usually show much larger pores than those observed here. For example, for MAG2 neutron scattering gave an inner (water pore) radius of 18.5 Å [14], cryo-EM gave 40 Å [15], dye-leakage in GUVs gave 14–36 Å depending on concentration [41], and confocal laser spectroscopy in cells gave 14 Å in bacteria and over 115 Å in mammalian cells [42]. Dye-leakage experiments gave a radius of 10 Å for PGLa-MAG [10] and 10–15 Å for MG-H2 [32]. The largest inner radius we have transiently observed in the present simulations is ~10 Å. Much of the discrepancy could be explained by the oligomeric state considered: experimental evidence determines that four peptides are the minimum component set for pore formation [14]. It is very likely that more peptides will be needed to stabilize larger pores. It might be that our observations correspond to “nascent” pores that could readily grow upon addition of further monomers. One technical concern here is possible limitations from the simulation box size. Three of the six systems were simulated in a larger box. However, other parameters were also varied at the same time and thus it is not possible to draw a conclusion on the effect of box size. Note that system 3 (PGLa tetramer) gave a small pore despite the presence of a larger box. Eventually, this issue will have to be resolved by simulations of larger peptide oligomer numbers in larger boxes. Because of the irregular arrangement of the peptides, we have been only able to measure approximate outer pore radii (“contact radii”) as the distance between the amino acids furthest apart in the pore. MAG2 homotetramers display the smallest outer radius (18 Å for the parallel tetramer, and 22 Å for the antiparallel one), followed by the PGLa-MAG2 systems (25–27 Å), PGLa (29 Å), and MG-H2 (33 Å).

Experimental results on pore structure are significant but scarce and do not provide a high-resolution structure. Inner and outer pore diameters from neutron scattering and the relative amounts of peptide and lipid suggested that magainin peptides form toroidal pores [14], as does melittin [43]. Oriented CD showed a change in orientation from surface to inserted at P/L ~1:30 to 1:10 in DMPC/DMPG 3:1 membranes, but only 30–80% of the peptides are oriented transmembrane within this concentration range [13]. In oriented CD one cannot distinguish between tilted orientations and a mixture of surface and transmembrane orientations. In the present simulations we observe mostly tilted peptides. Melittin in comparison, studied by a similar computational approach, appears much more likely to adopt I-state orientations [44]. The present results support the suggestion that magainin forms toroidal, rather than barrel-stave, pores. The number of headgroups observed to enter the bilayer area increased with pore size, as one would expect. The average helicity of the peptides over the whole simulation varies between 50–77%, and these values are in the range determined by CD of MAG2 amide upon binding POPC/POPG 3:1 membranes [45]. The loss of helical content in the C-terminal region of MAG2 has been previously reported in spectroscopy studies [46]. The average helicity of the four monomers during the MG-H2 simulation is higher than that reported in a previous study [31]. For the MAG2 tetramers significantly higher helicity is observed in the antiparallel system (Table 2).

In the case of PGLa, valuable results have been obtained in recent years from solid-state NMR [47]. At low P/L ratio the peptide adopts a surface orientation [23], as does MAG2 [6]. At P/L ratio 1:50 or higher the peptide shifts to a T-state, with a 120° tilt angle and the C-termini toward the membrane interior [24,48], which was suggested to result from formation of an antiparallel dimer. However, simulations of dimers on the membrane surface failed to see this increase in tilt angle [49]. Interestingly, the orientation of the monomers in our PGLa simulation agrees very well with these findings. In the PGLa simulation, the overall orientation of the four monomers is almost identical with respect to the two leaflets of the membrane, so the stabilized final structure resembles a geometrically perfect funnel (Fig 5B). The average tilt angle values of the PGLa peptides in the simulation (Fig 2C) agree well with those obtained from solid-state NMR experiments at similar peptide concentration in DMPC/DMPG 3:1 (~60°/120°) and DMPC membranes (~125°) [23,24,48]. The MAG2 tetramer adopts a similar structure in one of the simulations (Fig 1), except it is not a perfect funnel and two of the peptides insert their N terminus into the membrane. Comparison between the two tetramers shows stabilizing intermolecular interactions involving E19 of MAG2, whereas no such interactions or loss of helical character occurs in PGLa. In the absence of such interactions, the spatial organization of PGLa is stabilized because it maximizes the exposure of the four lysine residues to the solvent.

Experimental evidence on the formation of dimers or higher oligomers of magainin peptides on the membrane surface is rather controversial (see S1 Text). Our implicit solvent results suggest that antiparallel dimers are more stable. Based on this, we have investigated the effect of the initial orientation of the peptides in the MAG2 tetramer on the final pore structure and stabilizing mechanism. In the parallel tetramer, the enhanced mobility observed at the beginning of the simulation leads to the rearrangement of the peptides towards an irregular final configuration (Fig 1B). The disordered distribution of the peptides in the final assembly facilitates the interaction between the negatively charged E19 and the lysine residues at positions 4, 10 and 11 (Fig 1C), and these interactions are further favored by the increased flexibility of the monomers termini due to the partial unfolding. In the case of the antiparallel tetramer, only a minimal helicity loss is observed in the N-terminus of one of the monomers (Fig 4B). Although the specific interactions established between the monomers are similar to those observed in the parallel tetramer, the final structure shows remarkable differences. The peptides are significantly less mobile and hence maintain the overall antiparallel orientation for the whole trajectory. The antiparallel tetramer shows a regular, almost symmetrical, structural arrangement supported by multiple pairwise interactions involving charged and aromatic residues (Fig 4B and 4C). Interestingly, the structure of these antiparallel dimers resembles two experimental structures [38,39] and solves the conundrum of how these structures could bind a lipid bilayer. This peptide distribution leads to a quite ordered pore structure and larger pore size, also favored by the presence of two monomers in an I-state conformation. As a common feature in both systems, the monomers align so they promote the interaction between the C-terminal E19 residue and the positively charged lysines in the N-terminus. In order to establish these favorable interactions, at least one of the peptides in the parallel tetramer moves towards an antiparallel-like orientation.

An important observation that could offer clues on the mechanism of action of the magainin peptides is the synergy between MAG2 and PGLa [10,25,26]. The fact that maximum synergy is observed at 1:1 ratio suggested the formation of a heterodimer [10]. Our implicit solvent modeling suggested plausible interactions between residues S8 and E19 in MAG2 and K12 and K19 in PGLa, which could make the antiparallel heterodimer more stable than the homodimer (see S1 Text). When these antiparallel dimers were simulated in all-atom bilayers, they lost the perfect alignment they had on the membrane surface but retained significant interactions between them (Fig 8). As observed in the implicit simulations, the most favorable interactions are established between E19 in MAG2 and the lysines in the C-terminal region of the PGLa peptides (average energy values of approximately -80 kcal/mol). E19 has been shown to be essential for maintaining the synergisitic effects of the PGLa-MAG2 in a previous mutagenesis study [10]. In both parallel and antiparallel simulations, we observed more favorable interaction energies between heteromeric than homomeric pairs. These results provide a satisfying explanation of the observed synergy, although further tests are needed.

A solid state NMR study of PGLa-MAG2 mixtures showed that in some lipid bilayers PGLa transitions to an inserted state but magainin remains on the surface [29]. It was suggested that magainin facilitates the insertion of PGLa by thinning the membrane, but a more recent study has shown that this transition takes place in membranes with positive spontaneous curvature, independently of their thickness [30]. The average hydrophobic thickness values in our simulations are close to that of unperturbed bilayers in the 120-lipids systems (~ 30 Å), and slightly smaller in the 71–80 lipid systems (25–28 Å). Therefore, membrane thinning is occurring mostly locally around the pore in our simulations. Nevertheless, we have not been able to assess the role of the membrane spontaneous curvature in our simulations. MAG2 and PGLa appear equally likely to adopt an I-state orientation. This might be because our P/L (1:18 or 1:30) is higher than that used in the experiments (1:50). There is some evidence that as P/L increases magainin tends to become more tilted [30].

In all the systems the interaction of the peptides is significantly stronger with the hydrophobic tails than with the headgroups of either DMPC or DMPG lipids. During the last microsecond of the six trajectories, we observe the formation of scattered hydrogen bonds involving the lysine residues in the N-terminus and center of the peptides and DMPG headgroups. However, these transient bonds are not long-lived, since the lysine residues are mainly engaged in peptide-peptide interactions. We do not find clustering of the DMPG lipids around the peptides or further stabilization of these in the membrane, and therefore, we cannot draw any definitive conclusion on the role of anionic lipids in the stability of the peptide orientations in the time-scale of our simulations. This issue will be studied more systematically in future work.

In summary, these long time-scale atomistic simulations have provided a plausible molecular picture for the tilted T-state identified by solid-state NMR experiments and a plausible mechanism for the synergy between MAG2 and PGLa. In addition, they show how an antiparallel peptide dimer can bind a membrane while at the same time promoting the formation of an aqueous pore. Future studies should explore the effect of oligomeric number on pore size and structure and a systematic study of the effect of lipid composition. Long simulations like those reported here seem able to locate and characterize local free energy minima corresponding to peptide-stabilized membrane pores. One challenge for future research will be to calculate the free energy of these states with respect to membrane surface-adsorbed peptides. A further challenge will be to develop a theory to explain the atomistic simulation results in terms of peptide sequence, lipid properties, and peptide-peptide, peptide-lipid interactions.

## Methods

A description of the systems simulated in the present study is given in Table 1. The initial configuration of the MG-H2 tetramer corresponds to the last structure of a 160-ns simulation carried out previously by our group [34]. All other tetramers were built from ideal α-helices as a tightly packed helical bundle with the hydrophobic face outwards. The helices in the bundle were oriented parallel to each other, except in systems 2 and 6, where the antiparallel dimers produced by implicit solvent modeling (see S1 Text) were replicated once to make a tetrameric bundle (the two dimers are parallel to each other). The helical bundles were placed parallel to the bilayer normal in the CHARMM-GUI server [50]. Equilibration was performed with NAMD [51] using the CHARMM C36 force field [52]. Harmonic constraints (k = 1 kcal/mol/Å^2^) were initially applied to waters, ions, phosphorus atoms, and the backbone of the peptides. An energy minimization was run for 20000 steps, followed by 7 ps of heating to 303 K. Constraints in the lipids, and in waters and ions were released in 100-ps and 500-ps sequential equilibration steps, respectively. Pressure control using the modified Nose-Hoover barostat with Langevin Dynamics was then activated, and the constraints in the peptides backbone were scaled down in decrements of 0.2 for 200 ps. A final unconstrained 1 ns equilibration step was performed. The integration time step for all equilibration runs was 1 fs. Lastly, a 5 to 10 ns production step was run in NAMD (time step 2 fs).

The production phase was run for 5–9 μs on the Anton Supercomputer. We used the program viparr to generate the input structure file from the topology file, and coordinates, velocities and extended system binary NAMD output files for each system. Long-range electrostatic calculations were carried out with the Gaussian Split Ewald method [53]. Accurate cut off values range between 10–13 were automatically calculated by the Anton setup protocol based on the chemical features of the systems. The particle motions, barostat and thermostat updates were carried out separately with the integration framework Multigrator [54]. Infrequent updates of both thermostat and barostat improve the performance of the simulation and numerical integration accuracy. In our simulation protocol, the Nose-Hoover thermostat was updated every 24 steps and the Martyna, Tuckerman and Klein (MTK) barostat was updated every 240 steps. The integration time step was set to 2 fs and frames were saved every 1080 picoseconds.

VMD was used for the visualization of the trajectories and format conversion [55]. CHARMM software was used for the calculation of pore radius and tilt angle values. Average interaction energies over the last microsecond of the trajectories were calculated with the ENER command in CHARMM [56]. The calculation of the number of headgroups and water molecules inside the bilayer has been carried out in the area delimited by the phosphate groups in the upper and lower leaflet, and the hydrophobic area, respectively. The programs CPPTRAJ and PTRAJ in the Ambertools 12 package were used for secondary structure and hydrogen bond analysis [57]. The tilt angle is defined as the angle between the N-C helix vector and a unit vector in the positive z direction (which in the figures can be towards the upper or lower leaflet). Insofar as the +z direction is arbitrary, supplementary angles are equivalent, but for a given system, the tilt angle values for the different peptides show the relative direction of their N and C-termini.

## Supporting Information

S1 Text Implicit solvent studies of MAG2 and PGLa homo- and heterodimers. (DOC)

S1 Fig Average density profiles of the six simulated systems over the last microsecond of the trajectory. The colors on this plot correspond to the colors of the peptides in Figs 1 and 4–8.
(JPG)

S2 Fig Association of antiparallel MAG2 peptides. Side by side comparison of the overall orientation of an antiparallel MAG2 dimer resolved by NMR [38] (A), and the final structure of the antiparallel MAG2 tetramer simulation (B). Residue E19 is shown as sticks to mark the position of the C-terminus in each peptide.
(JPG)
