# Supplementary material for: Pore Structure and Synergy in Antimicrobial Peptides of the Magainin Family
Source: PLoS Comput Biol. 2016 Jan 4;12(1):e1004570. doi: 10.1371/journal.pcbi.1004570 (PMC4699650; doi:10.1371/journal.pcbi.1004570)
Supplement: S1 Text — (DOC) [file pcbi.1004570.s001.doc]

**Pore structure and synergy in antimicrobial peptides**

**of the magainin family**

Almudena Pino-Angeles, John M. Leveritt III, Themis Lazaridis*

Department of Chemistry, The City College of New York,

160 Convent Avenue, New York, NY, 10031, USA

**S1 Appendix. Comparison of the stability of dimers using implicit membrane modeling**

A plausible explanation for the magainin-PGLa synergy is the formation of a stable heterodimer between magainin and PGLa [10]. Crosslinking studies of Cys containing analogs suggested a parallel structure for this heterodimer [27]. Homodimerization of magainin has been inferred from a sigmoidal membrane binding isotherm [40]. An antiparallel structure for this dimer was determined by Transfer NOE measurements in bilayers [38] and by crystallization of a mutant in a racemic mixture [39]. Homodimerization of PGLa has not been experimentally detected, although an antiparallel dimer was proposed to explain solid state NMR results [24]. All-atom simulations found an antiparallel dimer to be more stable than a parallel one [49]. An antiparallel arrangement was adopted based on energetic arguments in early modeling of the magainin pore [11].

Here we explore the relative stability of magainin and PGLa homo- and heterodimers on a 30% anionic membrane surface using the implicit membrane model IMM1 [S1,S2]. The starting configurations were obtained from simulations of the monomers, which resulted in an orientation parallel to the membrane with the hydrophobic side of the helix towards the membrane interior. Then, parallel and antiparallel dimers were constructed by translating and rotating the monomer structures. For homodimers there is one possible parallel configuration and two antiparallel configurations, “L” and “R”. For heterodimers there are two possible parallel and two antiparallel configurations (Fig. S1). In all cases the helix axes were initially placed 12 Å apart. Then, a 1-ns simulation was run restraining the center of mass of the two helices to be less than 12.5 Å apart. Average effective energies were calculated over the last 0.9 ns of this simulation. Unconstrained simulations were also performed.


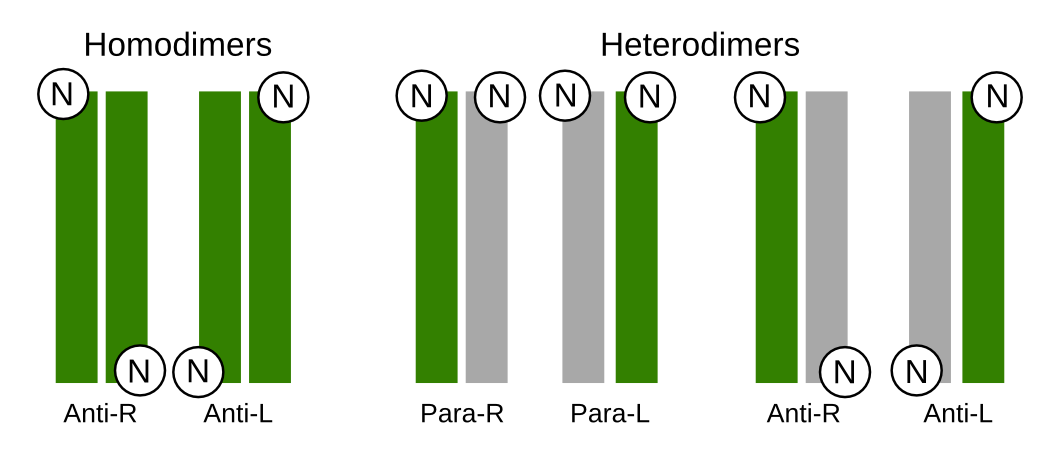


**Fig. S1. Possible dimer arrangements**

The resulting average energies are shown in Table S1. The energies of separate monomers are also shown to give an idea of the absolute stability of the dimers. For PGLa parallel and antiparallel structures have similar energy and modest stability, compared to separate monomers. In unconstrained simulations, the dimers most often either dissociated or shifted to form end-to-tail dimers. These results are understandable in terms of the sequence of PGLa, which does not provide many opportunities for salt bridge or polar interactions (there is only one Ser in addition to the 4 Lysines). Hydrophobic interactions are possible in solution, but less so on the membrane interface where most hydrophobic residues are buried in the membrane.

**Table S1. Average effective energies of dimers.**

| Dimer composition | Energy (kcal/mol) | Dimer composition | Energy (kcal/mol) |
| --- | --- | --- | --- |
| P + P | -823 ± 2 | PMpara-L | -908 ± 4 |
| M + M | -983 ± 3 | PMpara-R | -917 ± 3 |
| P + M | -903 ± 3 | PManti-L | -932 ± 2 |
| PPpar | -833 ± 2 | PManti-R | -928 ± 4 |
| PPanti -L | -831 ± 4 | HivHiiPara-L | -999 ± 3 |
| PPanti-R | -836 ± 2 | HivHiiPara-R | -995 ± 8 |
| MMpar | -999 ± 4 | HivHiiAnti-L | -1000 ± 3 |
| MManti-L | -1007 ± 6 | HivHiiAnti-R | -1004 ± 4 |
| MManti-R | -1022 ± 7 |  |  |

Hii and Hiv are the peptides used by Hara & Matsuzaki, corresponding to magainin and PGLa, respectively. The first three lines refer to two dissociated monomers.

Magainin homodimers are more stable, especially the antiparallel ones. The presence of a Glu, a His, an Asn and two Ser residues provide opportunities for salt bridge and hydrogen bond formation. Fig. S2, an energy minimized snapshot of the anti-R dimer, shows proximity of E19 with S8 of another monomer. Salt bridges between E19 and K4 on the other monomer are also occasionally formed during the dynamics. In the anti-L dimer E19 points away from the other monomer and has few opportunities for stabilizing interactions. In unconstrained simulations magainin dimers remain bound together, but the parallel and anti-L dimers do not bind to the membrane as tightly as anti-R.


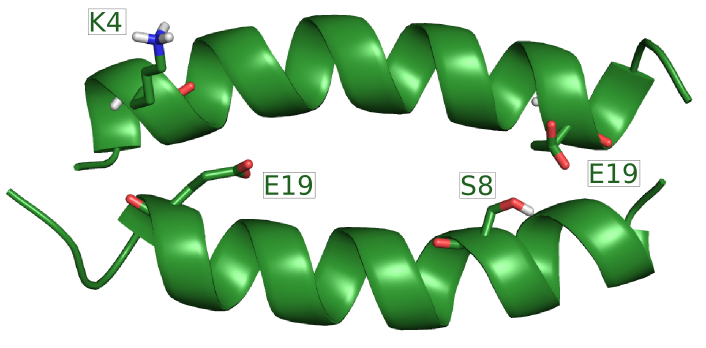


**Fig. S2. Snapshot from the magainin anti-R dimer simulation.**

The PGLa-magainin heterodimer is significantly more stable in an antiparallel configuration, consistent with previous modeling results [49]. The two antiparallel topologies have similar energies. An energy minimized snapshot of the anti-R dimer (Fig. S3) shows a salt bridge between magainin E19 and PGLa K12 and an hydrogen bond between magainin S8 and PGLa K19. These interactions are absent in the anti-L dimer, which derives its stability mostly from interactions between the termini. In unconstrained simulations the antiparallel dimers stay bound, whereas the parallel dimers often dissociate or move away from the membrane.


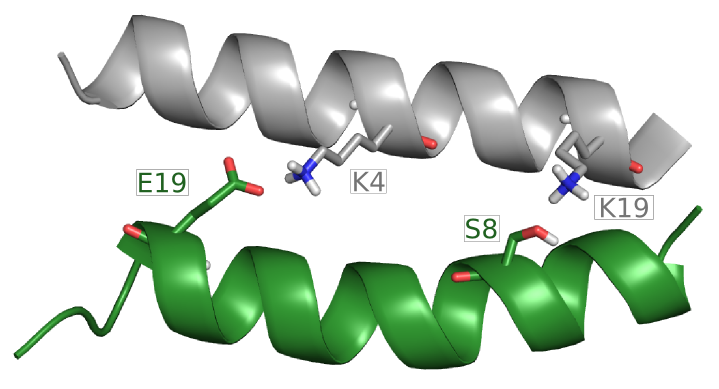


**Fig. S3. Snapshot from the PGLa-magainin anti-R dimer simulation.**

If we calculate the energy of the hypothetical reaction PP + MM → 2PM using the most favorable values for each dimer in Table S1, we obtain -6 ± 8 kcal/mol. Although the statistical uncertainty does not allow a definite conclusion, the heterodimer appears to be more stable than the homodimers, consistent with the observed synergy. A qualitative rationale for this can be obtained by examining the interactions in Figs. S2 and S3. The fact that both antiparallel heterodimers are energetically feasible could add entropic stability to the heterodimer. The importance of E19 for the magainin-PGLa synergy has been verified by mutagenesis [10].

As mentioned above, Hara *et al*. [27] concluded that the PGLa-magainin heterodimer is parallel. To resolve the discrepancy with the above calculations we also modeled the exact sequences used by these authors, which contained a Trp substitution, blocked termini, and a GGC addition at the C terminus (Table S1). We see that for these sequences the energies of parallel and antiparallel arrangements are very similar. The major contributor to this change is the reduced interaction between the termini.

**References**

S1. Lazaridis T. Effective energy function for proteins in lipid membranes. Proteins. 2003;52(2):176–92.

S2. Lazaridis T. Implicit solvent simulations of peptide interactions with anionic lipid membranes. Proteins. 2005;58(3):518–27.
