# Supplementary figures and images for: Pore Structure and Synergy in Antimicrobial Peptides of the Magainin Family
Source: PLoS Comput Biol. 2016 Jan 4;12(1):e1004570. doi: 10.1371/journal.pcbi.1004570 (PMC4699650; doi:10.1371/journal.pcbi.1004570)

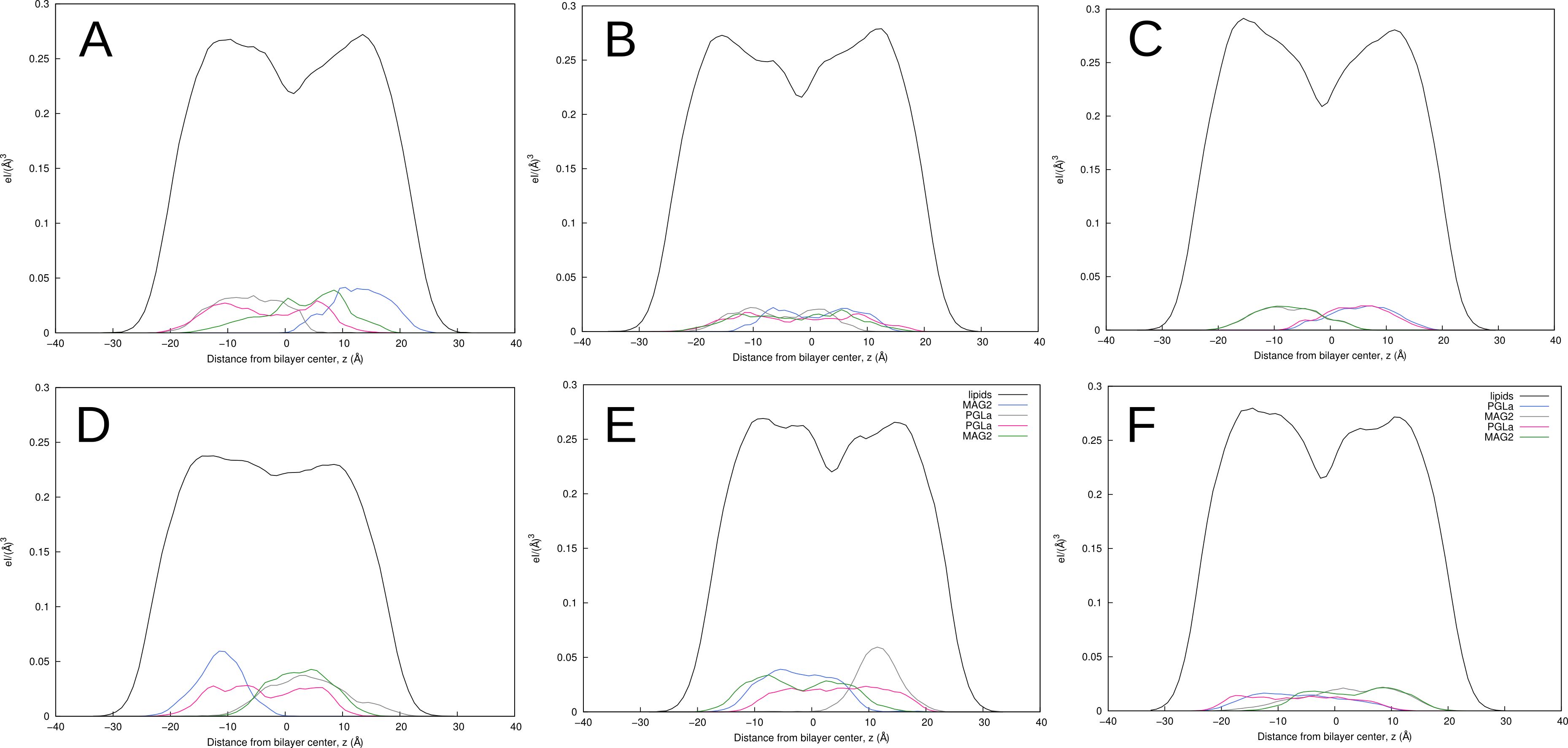

Supplement: S1 Fig — The colors on this plot correspond to the colors of the peptides in Figs 1 and 4–8. (JPG) [file pcbi.1004570.s002.jpg]

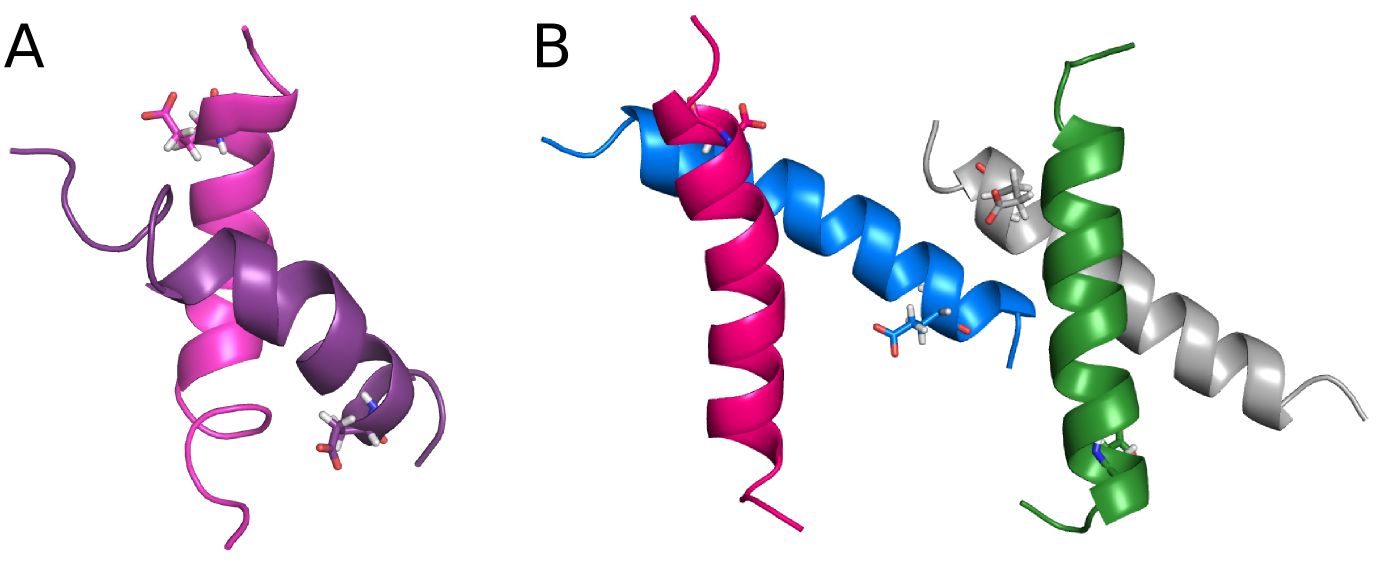

Supplement: S2 Fig — Side by side comparison of the overall orientation of an antiparallel MAG2 dimer resolved by NMR [38] (A), and the final structure of the antiparallel MAG2 tetramer simulation (B). Residue E19 is shown as sticks to mark the position of the C-terminus in each peptide. (JPG) [file pcbi.1004570.s003.jpg]
